# Minimum Winfree loop determines self-sustained oscillations in excitable Erdös-Rényi random networks

**DOI:** 10.1038/s41598-017-06066-6

**Published:** 2017-07-18

**Authors:** Yu Qian, Xiaohua Cui, Zhigang Zheng

**Affiliations:** 1 Nonlinear Research Institute, Baoji University of Arts and Sciences, Baoji, 721007 China; 2 School of Systems Science, Beijing Normal University, Beijing, 100875 China; 3 Institute of Systems Science, Huaqiao University, Xiamen, 361021 China; 4 College of Information Science and Engineering, Huaqiao University, Xiamen, 361021 China

## Abstract

The investigation of self-sustained oscillations in excitable complex networks is very important in understanding various activities in brain systems, among which the exploration of the key determinants of oscillations is a challenging task. In this paper, by investigating the influence of system parameters on self-sustained oscillations in excitable Erdös-Rényi random networks (EERRNs), the minimum Winfree loop (MWL) is revealed to be the key factor in determining the emergence of collective oscillations. Specifically, the one-to-one correspondence between the optimal connection probability (OCP) and the MWL length is exposed. Moreover, many important quantities such as the lower critical connection probability (LCCP), the OCP, and the upper critical connection probability (UCCP) are determined by the MWL. Most importantly, they can be approximately predicted by the network structure analysis, which have been verified in numerical simulations. Our results will be of great importance to help us in understanding the key factors in determining persistent activities in biological systems.

## Introduction

The brain system is composed of a large number of neurons. A single neuron possesses rich dynamical behaviors, such as periodic and even chaotic spiking and bursting. Moreover, the brain system can present multiple modes of persistent electrical oscillations at different levels^1–13^. Recent experimental studies have shown that the electroencephalography signals detected from the brain system comprise diverse oscillatory electrical activities. These oscillations, which can be roughly classified into several categories, such as delta, alpha, beta, and gamma oscillations, exist across a number of functional domains in the brain and are related to some specific and important physiological functions^14–23^. For example, it was found that phase entrainment of human delta oscillations can mediate the effects of expectation on reaction speed^20^. Bollimunta revealed the neuronal mechanisms of cortical alpha oscillations in awake-behaving macaques^21^. Kay exposed a beta oscillation network in the rat olfactory system during a 2-alternative choice odor discrimination task^22^. Palva discovered that the distinct gamma-band can evoke the responses to speech and non-speech sounds in humans^23^.

Since the studies of “small-world”^24^ networks proposed by Watts and Strogatz and “scale-free”^25^ networks proposed by Albert and Barabási, remarkable advances have been achieved in a lot of fields related to complex networks. Recently, the problems of self-sustained oscillations in excitable complex networks have become one of the central topics under investigation due to their extensive applications in brain systems. Exploring the key determinants of oscillations in these systems is a challenging task. Theoretically, diverse self-sustained oscillatory activities and related determining mechanisms are reported in different kinds of excitable complex networks^26–35^. For example, we discovered the one-dimensional (1D) Winfree loops to support self-sustained target group patterns in excitable small-world networks^31^. Liao *et al*. revealed the center nodes and small skeletons to sustain target wave like patterns in excitable homogeneous random networks^33^. Mi and collaborators exposed the mechanism of long-period rhythmic synchronous firings in excitable scale-free networks to explain the temporal information processing in neural systems^34^. Recently, we investigated the emergence of self-sustained oscillations in excitable Erdös-Rényi random networks (EERRNs)^35^. It was discovered that, at specific system parameters, periodical self-sustained oscillations can emerge in EERRNs in an appropriate connection probability interval, and there is an optimal connection probability (OCP) for supporting the oscillations. However, whether there is intrinsic mechanism in determining the oscillations in EERRNs, especially in determining the connection probability interval and the OCP for supporting the oscillations, is still unclear. In this paper, by investigating the influence of system parameters on self-sustained oscillations in EERRNs, the MWL is exposed to be the intrinsic mechanism in determining the emergence of collective oscillations. Furthermore, the one-to-one relationship between the OCP and the MWL length is revealed explicitly. More importantly, the connection probability interval (i.e., the lower critical connection probability (LCCP) and the upper critical connection probability (UCCP)) and the OCP for supporting the oscillations in EERRNs are exposed to be determined by the MWL. These three important quantities can be approximately predicted by the network structure analysis, and have been verified in numerical simulations.

## Model descriptions

In this paper, we adopt the Bär-Eiswirth model^36^ as node dynamics to construct the EERRN. The evolution of the studied network dynamics is described by the following equations:

1 $$\frac{{\rm{d}}{u}_{i}}{{\rm{d}}t}=-\frac{1}{\varepsilon }{u}_{i}({u}_{i}-\mathrm{1)}({u}_{i}-\frac{{v}_{i}+b}{a})+D\sum _{j=1}^{N}{A}_{i,j}({u}_{j}-{u}_{i}),$$

2 $$\frac{{\rm{d}}{v}_{i}}{{\rm{d}}t}=f({u}_{i})-{v}_{i}\mathrm{.}$$

Here *i* = 1, 2, …, *N* represents excitable nodes in the network. Variables *u*_*i*_ and *v*_*i*_ describe the activator and the inhibitor of the *i*th node, respectively. The function *f*(*u*) takes the following form:

3 $$f(u)=\{\begin{array}{cc}0 & u < \frac{1}{3},\\ 1-6.75u{(u-1)}^{2} & \frac{1}{3}\le u\le 1,\\ 1 & u > 1.\end{array}$$

The relaxation parameter $\varepsilon \ll 1$ represents the time ratio between the activator *u* and the inhibitor *v*. The dimensionless parameters *a* and *b* denote the activator kinetics of the local dynamics and can effectively control the excitation threshold (the excitation threshold of Bär-Eiswirth model is determined by *u*_T_ = *b*/*a*). *D* is the coupling strength which decides the interaction intensity between linking nodes. *A*_*i*,*j*_ is the adjacency matrix element, and is defined as *A*_*i*,*j*_ = *A*_*j*,*i*_ = 1 if there is a bidirectional connection linking nodes *i* and *j*, and *A*_*i*,*j*_ = *A*_*j*,*i*_ = 0 otherwise. The connections between every pair of nodes in the ERRN are linked with a probability *P*. Consequently, the total number of connections is expected to be *PN*(*N* − 1)/2. Here we should mention that, by manipulating the connection probability *P*, one can produce a number of EERRNs with the same *P*. For a given *P*, there are a lot of network realizations. Numerically, eqs (1) and (2) are integrated by the forward Euler integration scheme with time step Δ*t* = 0.02. The random initial condition is used in the numerical simulation (i.e., the initial variables *u*_*i*_(*t* = 0) and *v*_*i*_(*t* = 0) are randomly given between 0 and 1).

In order to reveal the key determinants of self-sustained oscillation, the oscillation proportion *p*_os_ is introduced as our order parameter to quantitatively investigate the influence of system parameters on self-sustained oscillations in EERRNs, which is defined as:

4 $${p}_{{\rm{os}}}=\frac{{N}_{{\rm{os}}}}{{N}_{{\rm{ALL}}}}\mathrm{.}$$

Here *N*_ALL_ is the total number of numerical simulations starting from random initial conditions for each set of parameters, and *N*_os_ is the number of self-sustained oscillations counted in *N*_ALL_ dynamical processes. To judge whether self-sustained oscillation emerges in the EERRN or not, we execute 400 time units for each simulation. If one of the nodes in the network executes persistent oscillatory cycles in the last 200 time units, self-sustained oscillation is deemed to emerge in EERRN. If nodes in the network are all in the rest state in the last 200 time units, no self-sustained oscillation can be observed in this numerical simulation. For each set of parameters, five thousand independent numerical simulations are performed (i.e., *N*_ALL_ = 5000). The above criterion is utilized to count the number of self-sustained oscillations *N*_os_ observed in this *N*_ALL_ = 5000 independent samples. And we will use the oscillation proportion as an order parameter to investigate the influence of system parameters on self-sustained oscillations in EERRNs and to reveal the key determinants.

## Numerical Results and Discussions

**Figure 1:**
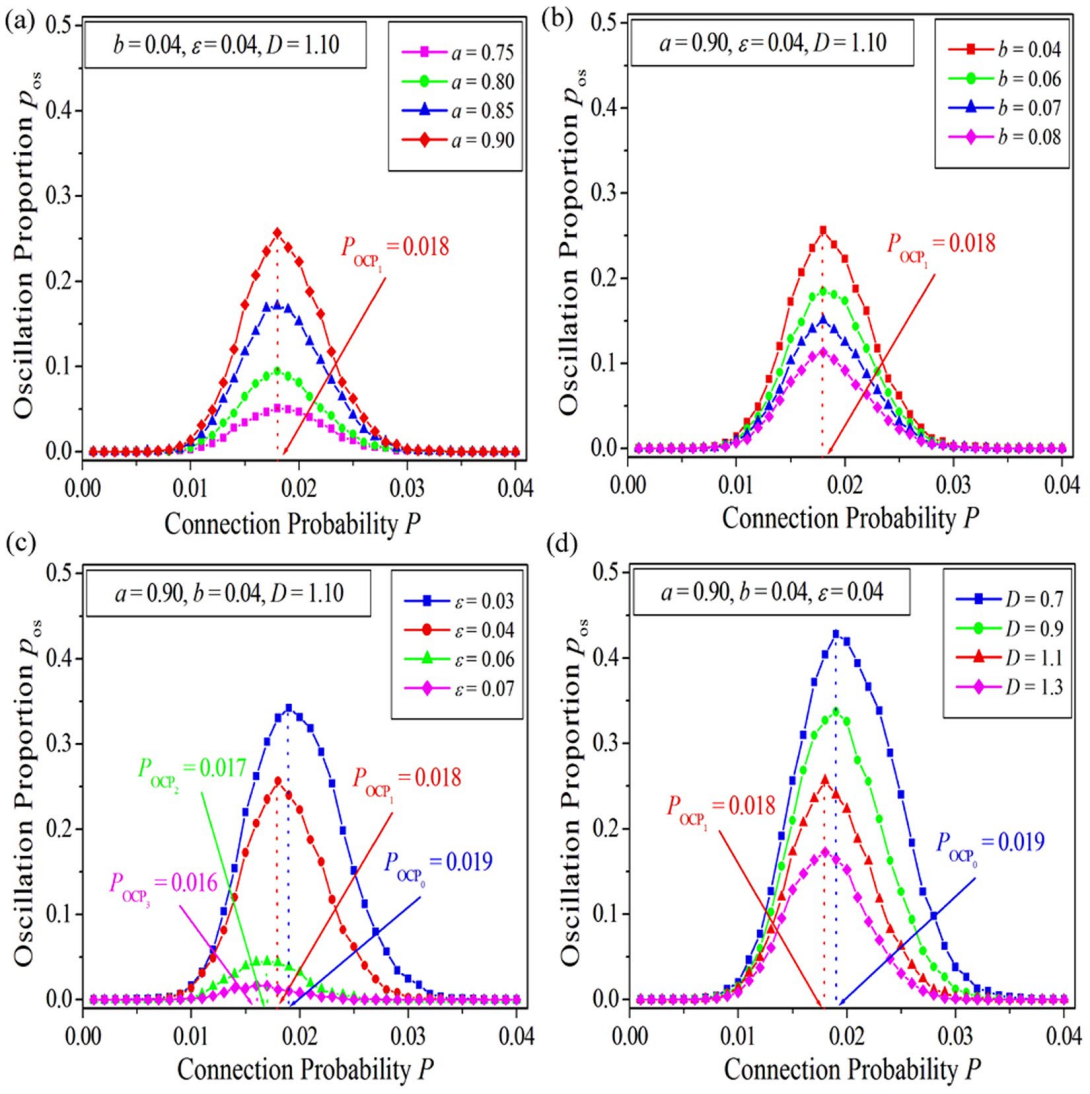
The dependence of the oscillation proportion *p*_os_ on the connection probability *P* at different system parameters *a* (**a**), *b* (**b**), *ε* (**c**) and *D* (**d**) in excitable Erdös-Rényi random networks (EERRNs). Other parameters are fixed and marked in the corresponding panels. The system size is selected as *N* = 100. The oscillation proportion is defined as ${p}_{{\rm{os}}}=\frac{{N}_{{\rm{os}}}}{{N}_{{\rm{ALL}}}}$. Here *N*_ALL_ is the total number of numerical simulations starting from random initial conditions for each set of parameters, and *N*_os_ is the number of self-sustained oscillations counted in *N*_ALL_ dynamical processes. For each set of parameters, five thousand independent numerical simulations are performed (i.e., *N*_ALL_ = 5000). The optimal connection probabilities (OCPs) *P*_OCP_ for supporting self-sustained oscillations in EERRNs at different system parameters are indicated.

In this part, we first perform a systematic investigation of the influence of system parameters on self-sustained oscillations in EERRNs. Figure 1(a–d) display the dependence of the oscillation proportion *p*_os_ on the connection probability *P* at different system parameters *a*, *b*, *ε* and *D* in EERRNs, respectively. Other parameters are fixed and marked in the corresponding panels. The system size is selected as *N* = 100. It is shown from Fig. 1(a) that, for a given *a* (such as *a* = 0.75, shown by pink squares), the EERRN can exhibit self-sustained oscillation in an appropriate connection probability interval (called as the oscillation parameter region), and will evolve into the homogeneous rest state at smaller *P* or larger *P*. As connection probability is increased in the oscillation parameter region, the oscillation proportion *p*_os_ first increases, then passes through a maximum, and finally decreases for larger *P*, which implies an OCP for supporting self-sustained oscillations in EERRNs (indicated by ${P}_{{{\rm{OCP}}}_{1}}=0.018$ in Fig. 1(a)). When *a* is increased (as shown by pink squares for *a* = 0.75, green dots for *a* = 0.80, blue triangles for *a* = 0.85 and red diamonds for *a* = 0.90), the oscillation proportion curve, which is located in the oscillation parameter region, ascends gradually. This means that the number of self-sustained oscillations emerging in EERRNs will increase as *a* is increased. Moreover, all the OCPs obtained for each oscillation proportion curve are approximately located at ${P}_{{{\rm{OCP}}}_{1}}=0.018$. This implies that the OCP for supporting self-sustained oscillations in EERRNs is independent of the system parameter *a*. From Fig. 1(a) we can find the parameter *a* can only influence the oscillation proportion *p*_os_, and has no effect on the OCP. The study of the relation between the oscillation proportion *p*_os_ and the connection probability *P* for different parameters *b* is shown in Fig. 1(b). It indicates that the parameter *b* can only influence the oscillation proportion *p*_os_ too. Specifically, *p*_os_ in the oscillation parameter region decreases as *b* is increased, and the OCPs obtained for different parameters *b* are all still located at ${P}_{{{\rm{OCP}}}_{1}}=0.018$.

Figure 1(c) reveals the dependence of the oscillation proportion *p*_os_ on the relaxation parameter *ε*. It is shown that, as *ε* is increased from 0.03 (shown by blue squares) to 0.07 (shown by pink diamonds) gradually, the oscillation proportion *p*_os_ decreases remarkably. Furthermore, the OCP decreases from ${P}_{{{\rm{OCP}}}_{0}}=0.019$ (corresponding to *ε* = 0.03) to ${P}_{{{\rm{OCP}}}_{3}}=0.016$ (corresponding to *ε* = 0.07) continuously. This surprising result indicates that the relaxation parameter *ε* can not only influence the number of self-sustained oscillations emerging in EERRNs, but also have an impact on the OCP for supporting the oscillations, which is distinct from the relation between *p*_os_ and parameters *a* and *b*. Figure 1(d) displays the dependence of the oscillation proportion *p*_os_ on the coupling strength *D*. Similar to the *p*_os_ ~ *P* relation obtained for the relaxation parameter *ε*, by increasing the coupling strength *D* from 0.7 (shown by blue squares) to 1.3 (shown by pink diamonds), the *p*_os_ decreases distinctly, and the OCP also decreases gradually from ${P}_{{{\rm{OCP}}}_{0}}=0.019$ (corresponding to *D* = 0.7 and *D* = 0.9) to ${P}_{{{\rm{OCP}}}_{1}}=0.018$ (corresponding to *D* = 1.1 and *D* = 1.3).

**Figure 2:**
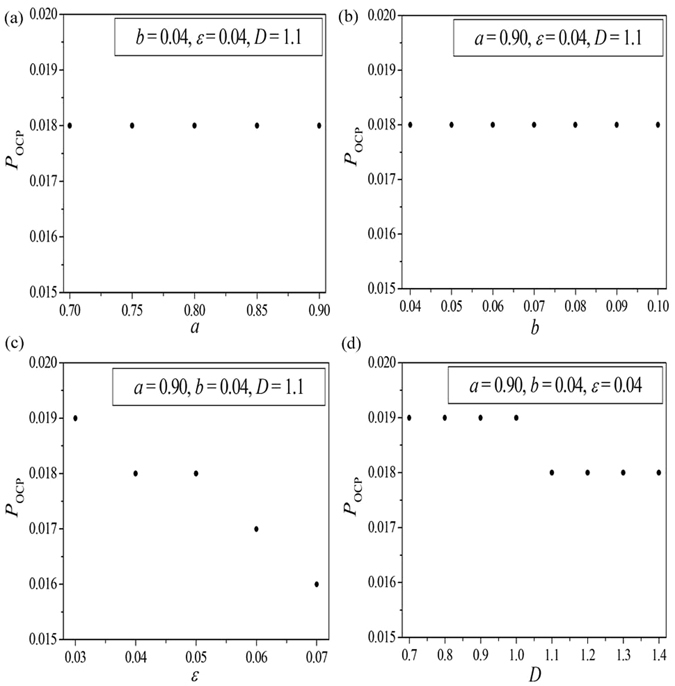
The relationship between the optimal connection probability *P*_OCP_ and the system parameters *a* (**a**), *b* (**b**), *ε* (**c**) and *D* (**d**). Other parameters are fixed and marked in the corresponding panels.

To intuitively expose the dependence of the optimal connection probability on system parameters, the relationships between the *P*_OCP_ and the parameters *a*, *b*, *ε* and *D* are shown in Fig. 2(a–d), respectively. It is shown from Fig. 2(a,b) that the optimal connection probabilities are all fixed at 0.018 for various choices of parameters *a* and *b*. Figure 2(c) shows the dependence of the optimal connection probability *P*_OCP_ on the relaxation parameter *ε*. As *ε* is increased from 0.03 to 0.07 continuously, the *P*_OCP_ decreases gradually from 0.019 (corresponding to *ε* = 0.03) to 0.016 (corresponding to *ε* = 0.07). The relationship between the *P*_OCP_ and the coupling strength *D* is shown in Fig. 2(d). As *D* is increased from 0.7 to 1.4 gradually, the *P*_OCP_ decreases from 0.019 (for 0.7 ≤ *D* ≤ 1.0) to 0.018 (for 1.1 ≤ *D* ≤ 1.4) slightly.

The above findings on the emergence of collective self-sustained oscillations and the dependence of collective oscillations on the parameters of the system indicate non-trivial behaviors exhibited in EERRNs. To explain the nonlinear dependence of the oscillation proportion curves on various system parameters shown in Fig. 1 and the relationship between the *P*_OCP_ and the parameters shown in Fig. 2, one should first clarify the following two key points. The first point is the influence of system parameters on the number of self-sustained oscillations emerging in EERRNs (i.e., the variation of *p*_os_ on system parameters). The second is the influence of system parameters on the OCP for supporting the oscillations in EERRNs (i.e., the variation of *P*_OCP_ on system parameters). Several conclusions that have been revealed in previous works^31, 35, 37^ can be applied to the interpretation of these two points. It is well known that the excitable wave propagating unidirectionally along an excitable loop can form one-dimensional Winfree loop^37^. Furthermore, it has been discovered in refs 31, 35 that, the formation of 1D Winfree loop, which exists as the oscillation source, is the key mechanism for maintaining self-sustained oscillation in excitable complex networks. Moreover, as we know, nodes in the excitable complex network must be excited in sequence. As a result, the excitable wave must propagate forward along the shortest path in the network. The 1D Winfree loop should also obey this shortest path rule. This means that the length of 1D Winfree loop should be as short as possible (i.e., the number of nodes in 1D Winfree loop should be as small as possible). However, due to the existence of the refractory period of excitable dynamics, the 1D Winfree loop cannot self-organize to a too small size topological loop. Therefore, there must be a MWL at a given set of system parameters. From the above discussions we can speculate that the MWL will play a key role in determining the self-sustained oscillation in EERRNs. Consequently, the relationship between the MWL and the system parameters is the crucial point to explain the results obtained in the above section.

**Figure 3:**
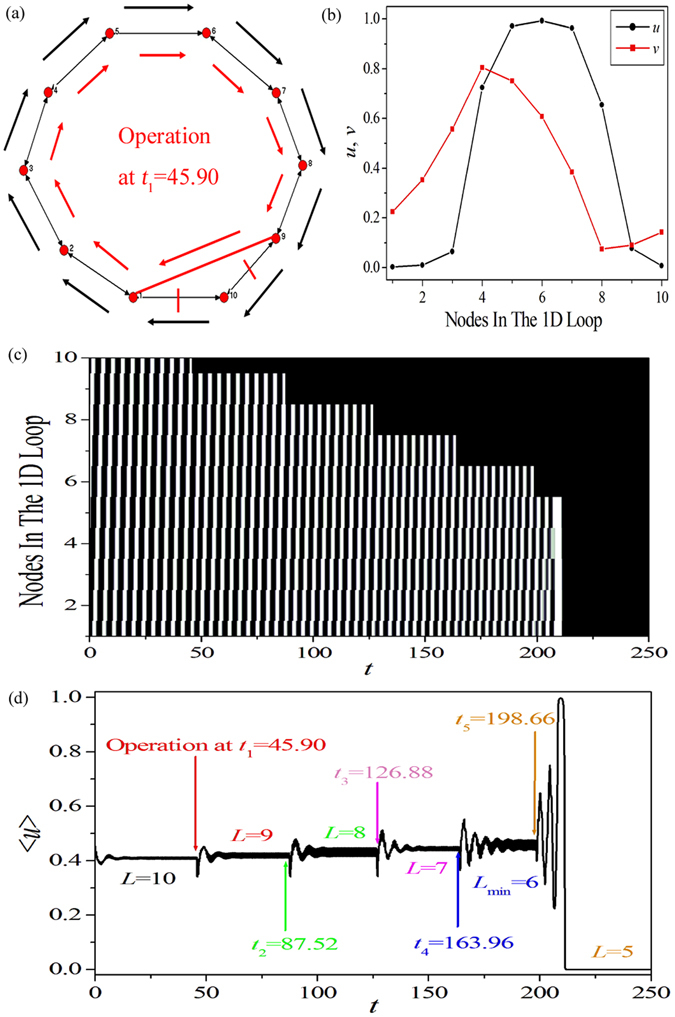
The schematic diagram of calculating the minimum Winfree loop (MWL) length *L*_min_. System parameters are chosen as *a* = 0.90, *b* = 0.04, *ε* = 0.04, and *D* = 1.1. (**a**) An artificial one-dimensional (1D) periodic excitable ring containing 10 nodes is constructed and the removal procedure (see below). (**b**) The wave form on this 1D periodic excitable ring, which is used as the initial condition for (**a**). When the wave form shown in (**b**) is applied, the excitable wave can propagate unidirectional along the pathway 1 → 2 → 3 → 4 → 5 → 6 → 7 → 8 → 9 → 10 → 1 to form a 1D Winfree loop in (**a**) (indicated by the outside black arrowed lines). This original loop is shortened by removing nodes in the loop in order to obtain a loop with the shortest length that are able to support a stable self-sustained oscillation. For the present 10 node loop, we take the following procedure: when the peak of the wave passes through node 1 at *t*_1_ = 45.90, node 10 in (**a**) is discarded from the 1D excitable ring (indicated by the two red short lines), and a connection between nodes 1 and 9 is added (indicated by the red long line). Then, a new shorter 1D Winfree loop composed by 1 → 2 → 3 → 4 → 5 → 6 → 7 → 8 → 9 → 1 is formed and re-organizes to support the self-sustained oscillation (indicated by the inner red arrowed lines). (**c**,**d**) The spatiotemporal pattern (**c**) and the trajectory of the collective variable $\langle u(t)\rangle =\frac{1}{N}{\sum }_{i=1}^{N=10}{u}_{i}(t)$ (**d**) as the above operations are executed successively at *t*_1_ = 45.90, *t*_2_ = 87.52, *t*_3_ = 126.88, *t*_4_ = 163.96 and *t*_5_ = 198.66, respectively, when each time one more node in the loop is removed.

The schematic diagram of the way we calculate the MWL length *L*_min_ is exhibited in Fig. 3. System parameters are chosen as *a* = 0.90, *b* = 0.04, *ε* = 0.04, and *D* = 1.1. An artificial 1D periodic excitable ring containing 10 nodes is constructed and is shown in Fig. 3(a). Figure 3(b) displays the wave form on this 1D periodic excitable ring, and this will be used as the initial condition for Fig. 3(a). When the wave form shown in Fig. 3(b) is applied, a unidirectional wave propagation is formed along the pathway 1 → 2 → 3 → 4 → 5 → 6 → 7 → 8 → 9 → 10 → 1 to form a 1D Winfree loop to support the oscillation (indicated by the outside black arrowed lines in Fig. 3(a)). We try to shorten the original loop by removing nodes in the loop in order to obtain a minimum loop with the shortest length that are able to support a stable self-sustained oscillation. For the present 10 node loop, we take the following procedure. When the peak of excitable wave passes through node 1, node 10 in Fig. 3(a) is discarded from the 1D excitable ring, i.e., we discard all the connections to node 10 (denoted by the two red short lines), and a connection between nodes 1 and 9 is added (denoted by the red long line). The operation is executed at some moment, e.g., *t*_1_ = 45.90. Then, a new shorter 1D Winfree loop composed by 1 → 2 → 3 → 4 → 5 → 6 → 7 → 8 → 9 → 1 is formed and re-organizes to support the self-sustained oscillation (indicated by the inner red arrowed lines). Figure 3(c,d) show the spatiotemporal pattern and the trajectory of the collective variable $\langle u(t)\rangle =\frac{1}{N}{\sum }_{i=1}^{N=10}{u}_{i}(t)$ as the above operations are executed successively at *t*_1_ = 45.90, *t*_2_ = 87.52, *t*_3_ = 126.88, *t*_4_ = 163.96 and *t*_5_ = 198.66, respectively, when each time one more node in the loop is removed. When five nodes in the original loop are discarded, 〈*u*(*t*)〉 is found to damp to zero quickly and the system evolves into a homogeneous rest state. This indicates that the MWL length, that are able to self-organize to support a global self-sustained oscillation, is *L*_min_ = 6 for the present system parameters *a* = 0.90, *b* = 0.04, *ε* = 0.04, and *D* = 1.1. The above procedure of seeking for the MWL length can be applied to general cases with different parameters and different node dynamics.

**Figure 4:**
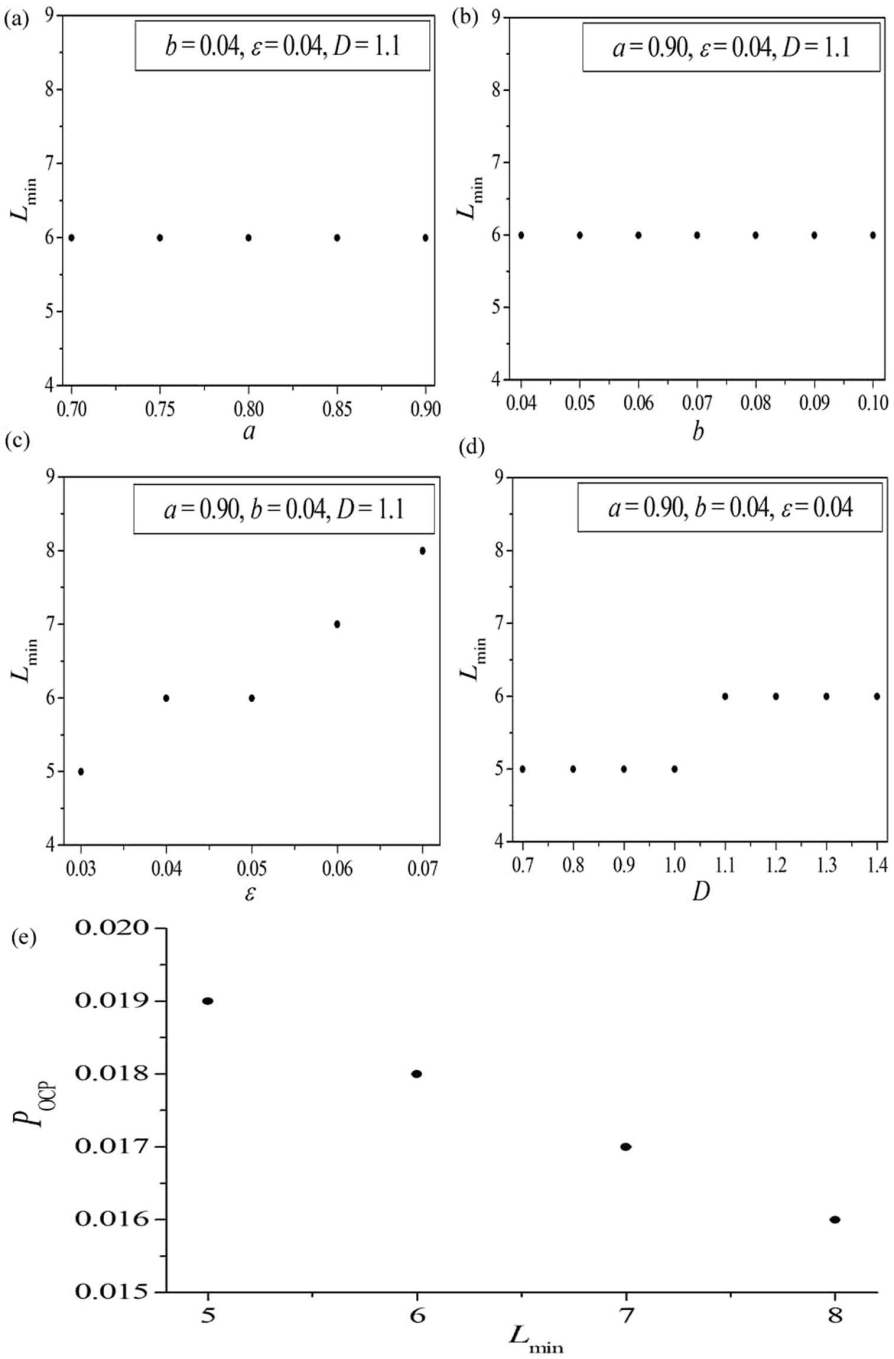
The relationship between the MWL length *L*_min_ and the system parameters *a* (**a**), *b* (**b**), *ε* (**c**) and *D* (**d**). Other parameters are fixed and marked in the corresponding panels. The MWL length *L*_min_ is calculated according to the schematic diagram illustrated in Fig. 3. (**e**) The dependence of the OCP *P*_OCP_ on the MWL length *L*_min_. It is obtained by comparing the results shown in Figs 2(a–d) and 4(a–d).

Figure 4(a–d) display the relationship between the MWL length *L*_min_ and the system parameters *a*, *b*, *ε* and *D*, respectively. Other parameters are fixed and marked in the corresponding panels. The MWL length *L*_min_ is calculated according to the schematic diagram illustrated in Fig. 3. By comparing the results shown in Figs 2(a–d) and 4(a–d), the dependence of the OCP *P*_OCP_ on the MWL length *L*_min_ can be obtained, and is shown in Fig. 4(e). It can be surprisingly found that, for a given *L*_min_, no matter what the specific parameters are, there is only one corresponding *P*_OCP_. Furthermore, the corresponding *P*_OCP_ decreases with increasing the MWL length *L*_min_. This means that the OCP for supporting self-sustained oscillations in EERRNs is determined by the MWL. Based on the results revealed in Fig. 4, now we can explain the influence of system parameters on self-sustained oscillations in EERRNs, especially in interpreting the variation of *p*_os_ and *P*_OCP_ on system parameters (shown by Figs 1 and 2).

For parameters *a* and *b*, the MWL lengths obtained are all fixed at *L*_min_ = 6 (shown in Fig. 4(a,b), respectively). As all *L*_min_ are the same for different *a* and *b*, it will result in a unique *P*_OCP_ corresponding to them. This is the reason why the OCP obtained for different parameters *a* and *b* are the same (located at ${P}_{{{\rm{OCP}}}_{1}}=0.018$, as shown in Fig. 1(a,b) or Fig. 2(a,b), respectively). Furthermore, as mentioned above, the excitation threshold of the Bär-Eiswirth model is determined by *u*_T_ = *b*/*a*. By increasing the parameter *a* or decreasing the parameter *b*, the excitation threshold of the local excitable dynamics will decrease, which improves the excitability of local excitable node and the wave propagation in excitable complex networks. This will result in the increase of the number of self-sustained oscillations in EERRNs. Consequently, the oscillation proportion *p*_os_ will increase as the excitation threshold is decreased, and the oscillation proportion curves shown in Fig. 1(a,b) can be observed.

Let us further discuss the oscillation proportion curves obtained for the relaxation parameter *ε* and the coupling strength *D*. These relations can be understood by the property of wave propagation in excitable media. As we know, the length of a given 1D Winfree loop *L* can be approximately calculated by the formula *L* ≈ *T* *V, where *T* is the oscillation period of the local excitable node and *V* is the propagating speed of the excitable wave along the 1D Winfree loop. Due to the existence of the refractory period of excitable dynamics, there is a minimum oscillation period *T*_min_, which approximately equals to the refractory period *T*_*f*_. Consequently, the length of MWL can be estimated approximately by the formula *L*_min_ ≈ *T*_min_*V ≈ *T*_f_*V. This indicates that the length of MWL is related to the refractory period of the local excitable node *T*_*f*_ and the propagating speed of the excitable wave *V*.

Figure 4(c) displays the dependence of the MWL length *L*_min_ on the relaxation parameter *ε*. It can be found that *L*_min_ increases as *ε* is increased. In Bär-Eiswirth model, $\varepsilon \ll 1$ is the relaxation parameter scale, which represents the time ratio between the activator *u* and the inhibitor *v*. It can be used to regulate the local excitable dynamics, especially in regulating the refractory period *T*_*f*_. Therefore, when the relaxation parameter *ε* is increased from 0.03 to 0.07 gradually, the refractory period *T*_*f*_ increases, which results in the increase of *L*_min_ from 5 (corresponding to *ε* = 0.03) to 8 (corresponding to *ε* = 0.07). On the other hand, a larger MWL length *L*_min_ means a more difficult formation of the 1D Winfree loop to support a stable self-sustained oscillation in the network. Consequently, the oscillation proportion *p*_os_ decreases remarkably, and the corresponding OCP also decreases from ${P}_{{{\rm{OCP}}}_{0}}=0.019$ (corresponding to *L*_min_ = 5) to ${P}_{{{\rm{OCP}}}_{3}}=0.016$ (corresponding to *L*_min_ = 8) continuously (shown in Fig. 1(c) or Fig. 2(c)). Figure 4(d) exhibits the relationship between the MWL length *L*_min_ and the coupling strength *D*. Similar to the dependence on the relaxation parameter *ε*, *L*_min_ increases slightly as *D* is increased. In excitable media, the coupling strength *D* is related to the propagation speed *V* of the excitable wave. Hence, as the coupling strength *D* is increased gradually, the propagation speed *V* of the excitable wave increases. This will lead to the increase of the MWL length *L*_min_ from 5 (for 0.7 ≤ *D* ≤ 1.0) to 6 (for 1.1 ≤ *D* ≤ 1.4) slightly, as shown in Fig. 4(d), which hinders the formation of the 1D Winfree loop for supporting self-sustained oscillations in EERRNs too. Consequently, the oscillation proportion *p*_os_ and the corresponding OCP decreases gradually, as shown in Fig. 1(d) or Fig. 2(d).

The above explorations reveal the intrinsic mechanism relating to the influence of system parameters on self-sustained oscillations in EERRNs. Phenomenally, the self-sustained oscillation in the EERRN seems to be decided by the system parameters. However, the one-to-one correspondence between the OCP and the MWL length given by Fig. 4(e) indicates that the emergence of collective oscillations in EERRNs is essentially determined by the MWL, which is hidden behind the system parameters. Therefore, the MWL is the key factor in determining the self-sustained oscillations in EERRNs.

The existence of the MWL is a key factor in leading to the self-sustained oscillation in EERRNs is manifested in the one-to-one relationship between the OCP and the MWL length. The underlying mechanism behind this one-to-one correspondence, or say, the existence of such MWL and its dominant role, however, is still unclear and needs to be further excavated. To solve this problem, the following two criteria, which are proposed in ref. 35 are adopted. (1) For a given set of system parameters, the network must contain a topological loop with a length that is not shorter than the MWL, i.e., *L* ≥ *L*_min_. (2) The average path length (APL) of a given network should satisfy *d*_APL_ ≥ *L*_min_ − 1.

Here we briefly interpret the main idea of these two criteria. Due to the existence of the refractory period of excitable dynamics, the 1D Winfree loop cannot self-organize to a too small size topological loop. This implies that there must be a MWL for a given set of system parameters (see the results shown in Fig. 4(a–d)). Consequently, criterion (1) (i.e., for the existence of a topological loop with *L* ≥ *L*_min_) is the necessary condition for the formation of 1D Winfree loop supporting self-sustained oscillations in EERRNs. When the connection probability *P* is small, the EERRN is largely sparse, criterion (1) can be used as the condition to expose the LCCP, beyond which self-sustained oscillations can emerge in EERRNs.

Now let us discuss criterion (2). If a MWL with a length *L*_min_ is formed, the diameter of this unidirectional 1D Winfree loop (along the wave propagation direction) is *L*_min_ − 1. This can be understood by resorting to the 1D Winfree loop shown in Fig. 3(a). For the artificial 1D periodic excitable ring with 10 nodes shown in Fig. 3(a), as a wave shown in Fig. 3(b) is applied, a unidirectional wave propagation is formed along the pathway 1 → 2 → 3 → 4 → 5 → 6 → 7 → 8 → 9 → 10 → 1, which forms a 1D Winfree loop to support the global oscillation. The diameter of this unidirectional 1D Winfree loop is 9. Moreover, it is well known that the APL of a network *d*_APL_ denotes the average shortest path between any two nodes in the network, which therefore can be approximately considered as the distance between the initially excited node to its corresponding driving node along the wave propagation path in the Winfree loop. If *d*_APL_ ≥ *L*_min_ − 1, the initially excited nodes in the network have enough time to response to the next excitation from their driving nodes. This may eventually form the 1D Winfree loop, and the self-sustained oscillation can emerge in the network. On the other hand, if *d*_APL_ < *L*_min_ − 1, the initially excited nodes are all in the refractory period. In this case, the 1D Winfree loop can not self-organize, and no oscillation can emerge in the network. Therefore, criterion (2) can be used as the condition to disclose the UCCP, below which self-sustained oscillations can emerge in EERRNs.

**Figure 5:**
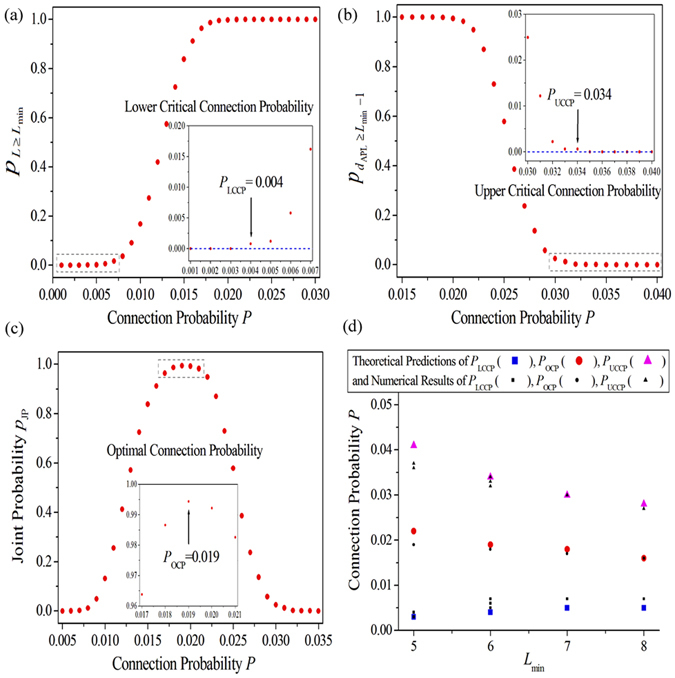
(**a**–**c**) The schematic diagram of analyzing the network structure in terms of the above two criteria to approximately predict the lower critical connection probability (LCCP), the OCP, and the upper critical connection probability (UCCP) for specific MWL. The MWL with the length *L*_min_ = 6 is used as example here. The insets is (**a**–**c**) display the local enlargement of the areas indicated by the grey dashed rectangles, respectively. The two blue dash lines in (**a**,**b**) are the critical values, which can help us approximately predict the LCCP and the UCCP. (**a**) The dependence of the proportion of network structures satisfying *L* ≥ *L*_min_ (that is defined as ${p}_{L\ge {L}_{{\rm{\min }}}}=\frac{{N}_{L\ge {L}_{{\rm{\min }}}}}{{N}_{{\rm{ALL}}}}$) on the connection probability *P* in ERRNs, here *N*_ALL_ is the total number of random network realizations performed for each connection probability *P*, and ${N}_{L\ge {L}_{\min }}$ is the number of network structures satisfys that possess a loop with the length *L* ≥ *L*_min_ in *N*_ALL_ independent samples. *N*_ALL_ = 5000 independent samples are performed for each connection probability *P*. (**b**) The dependence of the proportion of network structures with an APL *d*_APL_ ≥ *L*_min_ − 1 (that is defined as ${p}_{{d}_{{\rm{APL}}}\ge {L}_{{\rm{\min }}}-1}=\frac{{N}_{{d}_{{\rm{APL}}}\ge {L}_{{\rm{\min }}}-1}}{{N}_{L\ge {L}_{{\rm{\min }}}}}$) on the connection probability *P* in ERRNs. Here ${N}_{{d}_{{\rm{APL}}}\ge {L}_{{\rm{\min }}}-1}$ is the number of network structures satisfying *d*_APL_ ≥ *L*_min_ − 1 counted in ${N}_{L\ge {L}_{{\rm{\min }}}}$ samples. (**c**) The dependence of the joint probability (JP) (that is defined as ${p}_{{\rm{JP}}}={p}_{L\ge {L}_{{\rm{\min }}}}\ast {p}_{{d}_{{\rm{APL}}}\ge {L}_{{\rm{\min }}}-1}$) on the connection probability *P*. (**d**) The dependence of the theoretical predictions (shown by large coloured symbols) and the numerical results (shown by small black symbols) of *P*_LCCP_, *P*_OCP_ and *P*_UCCP_ on the MWL length *L*_min_.

Figure 5(a–c) illustrate the schematic diagram of the way that we analyze the network structure in terms of the above two criteria to approximately predict some important quantities such as the LCCP, the OCP and the UCCP for specific MWL. The MWL with the length *L*_min_ = 6 is used as our example here. The insets display the local enlargement of the areas indicated by the grey dashed rectangles in Fig. 5(a–c). The two blue dash lines in Fig. 5(a,b) are the critical values, which can help us approximately predict the LCCP and the UCCP. Figure 5(a) displays the dependence of the proportion of network structures satisfying *L* ≥ *L*_min_ (that is defined as ${p}_{L\ge {L}_{{\rm{\min }}}}=\frac{{N}_{L\ge {L}_{{\rm{\min }}}}}{{N}_{{\rm{ALL}}}}$) on the connection probability *P* in ERRNs, here *N*_ALL_ is the total number of random network realizations performed for each connection probability *P*, and ${N}_{L\ge {L}_{{\rm{\min }}}}$ is the number of network structures that possess a loop with the length *L* ≥ *L*_min_ in *N*_ALL_ independent samples. In our simulations *N*_ALL_ = 5000 independent ERRNs are performed for each connection probability *P*. It is shown that the ${p}_{L\ge {L}_{\min }}$ increases from 0 to 1 as the connection probability *P* is increased. More importantly, from the inset shown in the Fig. 5(a), we can find that the ${p}_{L\ge {L}_{\min }}$ can be larger than 0 as the connection probability *P* reaches 0.004. This means that the network structure in ERRNs can satisfy criterion (1) when the connection probability *P* ≥ 0.004. This naturally gives the LCCP *P*_LCCP_ = 0.004, beyond which self-sustained oscillations can emerge in EERRNs. Figure 5(b) shows the dependence of the proportion of network structures with an APL *d*_APL_ ≥ *L*_min_ − 1 (that is defined as ${p}_{{d}_{{\rm{APL}}}\ge {L}_{\min }-1}=\frac{{N}_{{d}_{{\rm{APL}}}\ge {L}_{\min }-1}}{{N}_{L\ge {L}_{\min }}}$) on the connection probability *P* in ERRNs. Here ${N}_{{d}_{{\rm{APL}}}\ge {L}_{{\rm{\min }}}-1}$ is the number of network structures satisfying *d*_APL_ ≥ *L*_min_ − 1 counted in ${N}_{L\ge {L}_{\min }}$ samples. It is shown that the ${p}_{{d}_{{\rm{APL}}}\ge {L}_{{\rm{\min }}}-1}$ decreases from 1 to 0 as the connection probability *P* is increased. More importantly, from the inset shown in the Fig. 5(b), we can find that the ${p}_{{d}_{{\rm{APL}}}\ge {L}_{{\rm{\min }}}-1}$ approaches 0 as the connection probability *P* is greater than 0.034. This means that the network structure in ERRNs fails to satisfy criterion (2) when the connection probability *P* > 0.034. This naturally gives the UCCP *P*_UCCP_ = 0.034, below which self-sustained oscillations can emerge in EERRNs. Figure 5(c) exhibits the dependence of the joint probability (JP) (that is defined as ${p}_{{\rm{JP}}}={p}_{L\ge {L}_{{\rm{\min }}}}\ast {p}_{{d}_{{\rm{APL}}}\ge {L}_{{\rm{\min }}}-1}$) on the connection probability *P*. The physical meaning of joint probability *p*_JP_ is the proportion of network structures satisfying both criterion (1) and criterion (2). It is shown that the joint probability *p*_JP_ first increases with increasing the connection probability *P*, and then decreases after passing a maximum. As we know, the more the network structure meets the above two criteria, the more the oscillation can emerge in EERRNs. That’s the reason why similar oscillation proportion curves shown in Fig. 1 can be obversed. More importantly, the peak of the JP curve locates at *P* = 0.019 (see the inset in Fig. 5(c)). This indicates that the number of network structures in ERRNs simultaneously satisfying criterion (1) and criterion (2) are the most at this connection probability. It can be used as the indicator to reveal the OCP (i.e., *P*_OCP_ = 0.019), at which most self-sustained oscillations can emerge in EERRNs.

Figure 5(d) reveals the dependence of the theoretical predictions (shown by large coloured symbols) and the numerical results (shown by small black symbols) of *P*_LCCP_, *P*_OCP_ and *P*_UCCP_ respectively on the MWL length *L*_min_. The theoretical predictions are performed according to the schematic diagram illustrated in Fig. 5(a–c). It is shown that numerical results are coincident with theoretical predictions very well. Here we should mention that, in our numerical simulation, we have observed multiple approximate values of *P*_LCCP_ and *P*_UCCP_ for a specific *L*_min_ (shown by numerical results in Fig. 5(d)). The reason is as follow: As shown by the two insets in Fig. 5(a,b), the ${p}_{L\ge {L}_{{\rm{\min }}}}$ and the ${p}_{{d}_{{\rm{APL}}}\ge {L}_{{\rm{\min }}}-1}$ obtained around the critical connection probabilities are very small (such as ${p}_{L\ge {L}_{{\rm{\min }}}}=0.0008$ for *P* = 0.004, ${p}_{L\ge {L}_{{\rm{\min }}}}=0.0012$ for *P* = 0.005, ${p}_{L\ge {L}_{{\rm{\min }}}}=0.0058$ for *P* = 0.006 in Fig. 5(a), and ${p}_{{d}_{{\rm{APL}}}\ge {L}_{{\rm{\min }}}-1}=0.0006$ for *P* = 0.034, ${p}_{{d}_{{\rm{APL}}}\ge {L}_{{\rm{\min }}}-1}=0.0006$ for *P* = 0.033, ${p}_{{d}_{{\rm{APL}}}\ge {L}_{{\rm{\min }}}-1}=0.0022$ for *P* = 0.032 in Fig. 5(b)). This means that the number of network structures in ERRNs satisfying the criterion (1) or the criterion (2) (i.e., the number of network structures, which satisfy the conditions for supporting the self-sustained oscillation in ERRNs) around the critical connection probabilities are very few. Furthermore, besides the existence of the network structure satisfying the conditions for supporting the self-sustained oscillation, the proper initial condition is another key point to form self-sustained oscillation. As the random initial condition is used in this paper, the randomness of initial condition can not ensure the emergence of self-sustained oscillation in these few network structures around these two critical connection probabilities, which are predicted by the criterion (1) and the criterion (2). This may result in the numerical results of *P*_LCCP_ and *P*_UCCP_ different (such as *P*_LCCP_ = 0.004, *P*_LCCP_ = 0.005, or even *P*_LCCP_ = 0.006 for the LCCP; *P*_UCCP_ = 0.034, *P*_UCCP_ = 0.033, or even *P*_UCCP_ = 0.032 for the UCCP). Consequently, for a given set of system parameters, a pair of *P*_LCCP_ and *P*_UCCP_, which are approximate to the predicted values, can be detected in numerical simulation. As a specific *L*_min_ can correspond to several sets of system parameters, we can observe multiple approximate values of *P*_LCCP_ and *P*_UCCP_ corresponding to a specific *L*_min_ in numerical simulation.

**Figure 6:**
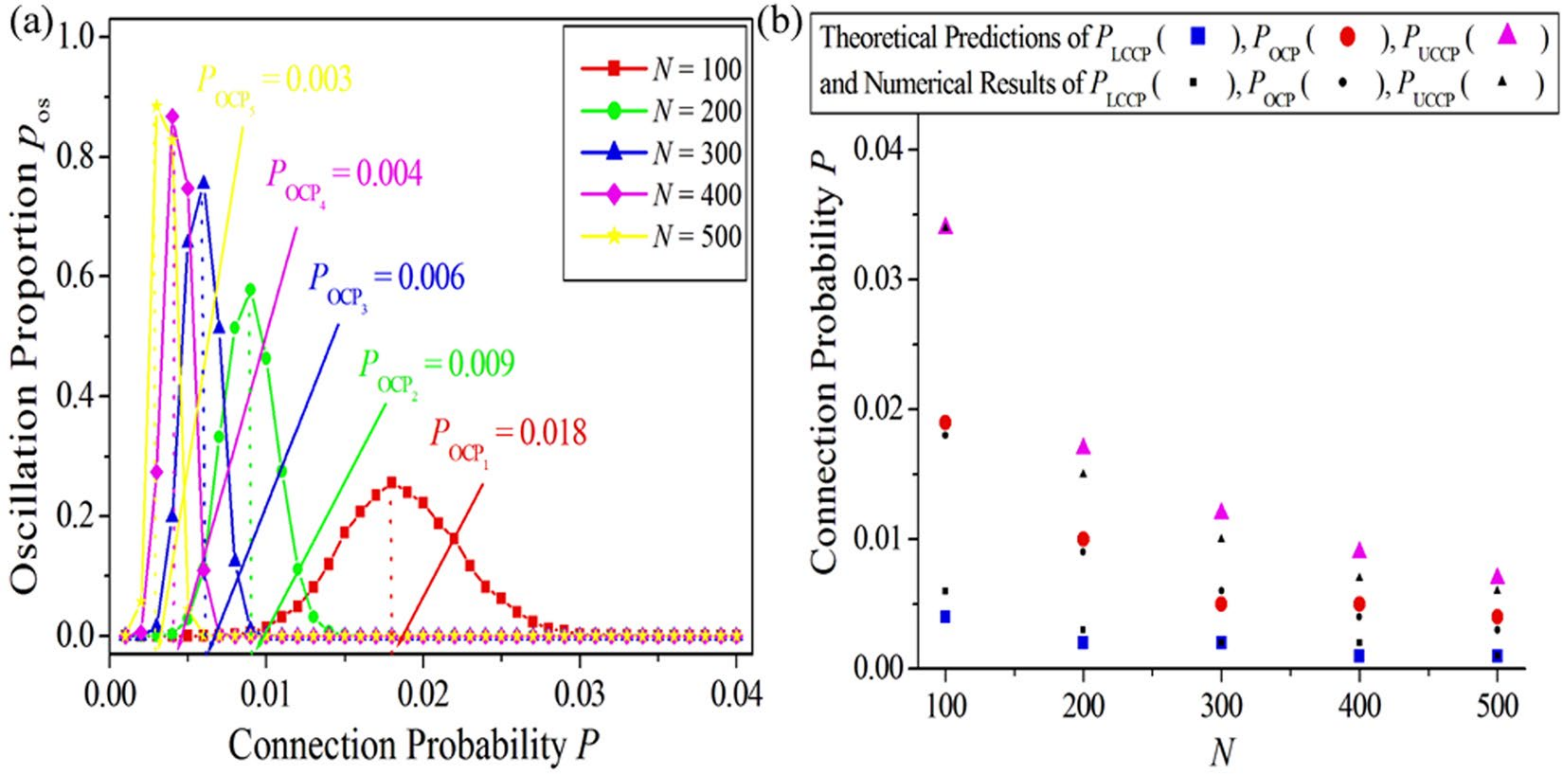
(**a**) The dependence of the oscillation proportion *p*_os_ on the connection probability *P* for different system sizes *N* in EERRNs. Here other parameters are chosen as *a* = 0.90, *b* = 0.04, *ε* = 0.04, and *D* = 1.1. *N*_ALL_ = 5000 independent randomly constructed excitable Erdös-Rényi networks are performed for each set of parameters. The OCPs *P*_OCP_ for each oscillation proportion curve are indicated. (**b**) The dependence of theoretical predictions (shown by large coloured symbols) and numerical results (shown by small black symbols) of *P*_LCCP_, *P*_OCP_ and *P*_UCCP_ on system size *N*.

System size is also an important ingredient in determining the spatiotemporal dynamics in EERRNs. Figure 6(a) presents the dependence of the oscillation proportion *p*_os_ on the connection probability *P* for different system sizes *N* in EERRNs (as shown by red squares for *N* = 100, green dots for *N* = 200, blue triangles for *N* = 300, pink diamonds for *N* = 400 and yellow stars for *N* = 500). Here other parameters are chosen as *a* = 0.90, *b* = 0.04, *ε* = 0.04, and *D* = 1.1. *N*_ALL_ = 5000 independent randomly constructed excitable Erdös-Rényi networks are performed for each set of parameters. The OCPs *P*_OCP_ for each oscillation proportion curve are indicated. It is shown that, when the system size *N* is increased gradually from 100 (shown by red squares) to 500 (shown by yellow stars), the oscillation proportion *p*_os_ significantly increases. Simultaneously, the OCP decreases from ${P}_{{{\rm{OCP}}}_{1}}=0.018$ to ${P}_{{{\rm{OCP}}}_{5}}=0.003$. This reveals that the system size *N* can not only affect the number of self-sustained oscillations emerging in EERRNs, but also impact on the OCP for supporting the oscillations. Figure 6(b) exhibits the dependence of theoretical predictions (shown by large coloured symbols) and numerical results (shown by small black symbols) of *P*_LCCP_, *P*_OCP_ and *P*_UCCP_ on system size *N*. It is shown that numerical results are in good agreement with theoretical predictions, which indicates that the analysis of the mechanism and consequent predictions revealed in this paper are independent of system size.

## Conclusion

In this paper, we explore the mechanism of self-sustained oscillation in EERRNs. To do this, we extensively investigated the influence of system parameters on self-sustained oscillations in EERRNs. An intuitive judgement is that the system parameters can not only influence the number of self-sustained oscillations emerging in EERRNs, but also impact the connection probability interval (i.e., the LCCP and the UCCP) and the OCP for supporting the oscillations. However, our investigation reveals that these phenomena are essentially determined by the MWL, which is deeply hidden behind the system parameters. The dependence of self-sustained oscillations emerging in EERRNs on system parameters can be interpreted by the MWL. Furthermore, the one-to-one correspondence between the OCP and the MWL length is revealed. Most importantly, the LCCP and the UCCP (i.e., the connection probability interval) and the OCP for supporting the oscillations in EERRNs are exposed to be determined by the MWL, and these three important quantities can be well predicted by network structure analysis, which have been verified in numerical simulations. The above explorations reveal the intrinsic mechanism relating to the influence of system parameters on self-sustained oscillations in EERRNs, and indicate that the MWL is the key factor in determining the collective oscillations in EERRNs. Finally, we disclose that the results revealed in this paper are independent of the system size.

Studies on issues related to self-sustained oscillations in excitable complex networks are very important, among which exploring the key determinants of oscillations is a challenging task. A systematic investigation of the MWL determined self-sustained oscillations in excitable Erdös-Rényi random networks and the related mechanism are expected to be useful both for theoretical understandings and practical applications. We hope our results can help us shed light on understanding the key factors in determining persistent activities in biological systems.
